# Supplementary material for: Effect of Nitric Oxide Pathway Inhibition on the Evolution of Anaphylactic Shock in Animal Models: A Systematic Review
Source: Biology (Basel). 2022 Jun 16;11(6):919. doi: 10.3390/biology11060919 (PMC9228251; doi:10.3390/biology11060919)
Supplement: Supplementary file 1 [file biology-11-00919-s001.zip › biology-1739200-supplementary.pdf]

Supplemental Figure S1. SYRCLE's risk of bias tool[22]. The excel sheet created to assess the risk of bias. Each row reflects a different paper. A summary is available in figure 4.

Pretreatment	1 was the allocation sequence adequately generated and applied? (were they randomised)	2 were the groups similar at baseline or were they adjusted for confounders in the analysis?	3 was the allocation adequately concealed?	4 were the animals randomly housed during the experiment?	5 were the caregivers &/or investigators blinded from knowledge which intervention each animal received during the experiment?	6 were animals selected at random for outcome assessment?	7 was the outcome assessor blinded?	8 were incomplete outcome data adequately addressed?				9 Are reports of the study free of selective outcome reporting?	10 was the study apparently free of other problems that could result in high risk of bias?	
								Were all animals included in the analysis?	Were the reasons for missing outcome data unlikely to be related to true outcome? (e.g., technical failure)	Are missing outcome data balanced in numbers across intervention groups, with similar reasons for missing data	Are missing outcome data imputed using appropriate methods?		a - Was the study free of contamination (pooling drugs)?	b - Was the study free of inappropriate influence of funders?
Osada et al.1994	Unclear	Yes	No	Unclear	No	Unclear	No	Yes	?	?	?	Yes	Yes	Unclear
Mitsuhata et al. 1995	Unclear	Yes	No	Unclear	No	Unclear	No	No	Yes	No	Yes	Yes	Yes	Yes
Shibamoto et al. 1996	Unclear	Yes	No	Unclear	No	Unclear	No	Yes	?	?	?	Yes	Yes	Yes
Bellou et al. 2003	Unclear	Yes	No	Yes	No	Unclear	No	Yes	?	?	?	Yes	Yes	Yes
Buzato et al. 2005	Unclear	Yes	No	Unclear	No	Unclear	No	Yes	?	?	?	Yes	Yes	Yes
Cauwels et al. 2006	Unclear	Yes	No	Yes	No	Unclear	No	Yes	?	?	?	Yes	Yes	Yes
Takano et al. 2007	Unclear	Yes	No	Yes	No	Unclear	No	Unclear	?			Yes	Yes	Yes
Zhang et al. 2009	Unclear	Yes	No	Yes	No	Unclear	No	Yes	?	?	?	Yes	Yes	Yes
Menardi AC et al. 2011	Unclear	Yes	No	Unclear	No	Unclear	No	Yes	?	?	?	Yes	Yes	Yes
Shinomiya et al. 2013	Unclear	Yes	No	Yes	No	Unclear	No	Yes	?	?	?	Yes	Yes	Yes
Albuquerque 2016	Unclear	Yes	No	Yes	No	Unclear	No	Yes	?	?	?	Yes	Yes	Yes
Mukai et al. 2018	Unclear	Yes	No	Yes	No	Unclear	No	Yes	?	?	?	Yes	Yes	Yes
Alburquerque AAS et al. 2020	Unclear	Yes	No	Unclear	No	Unclear	No	No	Unclear	No	Unclear	Yes	Yes	Yes
Albuquerque et al. 2022	Unclear	Yes	No	Unclear	No	Unclear	No	Yes	?	?	?	Yes	Yes	Yes
**Post treatment**														
Amir & English 1991	Unclear	Yes	No	Unclear	No	Unclear	No	Yes	?	?	?	Yes	Yes	Unclear
Mitsuhata et al. 1995	Unclear	Yes	No	Unclear	No	Unclear	No	Yes	?	?	?	Yes	Yes	Yes
Buzato et al. 2005	Unclear	Yes	No	Unclear	No	Unclear	No	Yes	?	?		Yes	Yes	Yes
Menardi AC et al. 2011	Unclear	Yes	No	Unclear	No	Unclear	No	Yes	?	?	?	Yes	Yes	Yes
Zheng F et al. 2013	Unclear	Yes	No	Yes	No	Unclear	No	Yes	?	?	?	Yes	Yes	Yes
Albuquerque 2016	Unclear	Yes	No	Yes	No	Unclear	No	Yes	?	?	?	Yes	Yes	Yes
Alburquerque AAS et al. 2020	Unclear	Yes	No	Unclear	No	Unclear	No	No	Unclear	No	Unclear	Yes	Yes	Yes
Albuquerque et al. 2022	Unclear	Yes	No	Unclear	No	Unclear	No	Yes	?	?	?	Yes	Yes	Yes
Yes	0	22	0	9	0	0	0	18	1	0	1	22	22	20
No	0	0	22	0	22	0	22	3	0	3	0	0	0	0
Unclear	22	0	0	13	0	22	0	1	2	0	2	0	0	2
?	0	0	0	0	0	0	0	0	19	19	19	0	0	0
